# Evolvability and robustness in populations of RNA virus Φ6

**DOI:** 10.3389/fmicb.2014.00035

**Published:** 2014-02-05

**Authors:** Daniel Goldhill, Angela Lee, Elizabeth S. C. P. Williams, Paul E. Turner

**Affiliations:** Department of Ecology and Evolutionary Biology, Yale UniversityNew Haven, CT, USA

**Keywords:** genetic robustness, evolvability, thermotolerance, bacteriophage, experimental evolution

## Abstract

Microbes can respond quickly to environmental disturbances through adaptation. However, processes determining the constraints on this adaptation are not well understood. One process that could affect the rate of adaptation to environmental perturbations is genetic robustness, the ability to maintain phenotype despite mutation. Genetic robustness has been theoretically linked to evolvability but rarely tested empirically using evolving populations. We used populations of the RNA bacteriophage ϕ6 previously characterized as differing in robustness, and passaged them through a repeated environmental disturbance: periodic 45°C heat shock. The robust populations evolved faster to withstand the disturbance, relative to the less robust (brittle) populations. The robust populations also achieved relatively greater thermotolerance by the end of the experimental evolution. Sequencing revealed that thermotolerance occurred via a key mutation in gene P5 (viral lysis protein), previously shown to be associated with heat shock survival in the virus. Whereas this identical mutation fixed in all of the independently evolving robust populations, it was absent in some brittle populations, which instead fixed a less beneficial mutation. We concluded that robust populations adapted faster to the environmental change, and more easily accessed mutations of large benefit. Our study shows that genetic robustness can play a role in determining the relative ability for microbes to adapt to changing environments.

## Introduction

Viruses are often capable of very rapid molecular evolution, allowing adaptation to new hosts, and other novel challenges (Duffy et al., 2008; Wasik and Turner, 2013). Although viruses can quickly adapt in response to environmental changes, possible constraints on viral adaptation are seldom studied (Burch and Chao, 2000; Turner and Elena, 2000). One trait that may affect the rate of adaptation in evolving populations is genetic robustness: the capacity to maintain phenotype despite perturbation from underlying mutations (de Visser et al., 2003; Wagner, 2008; Draghi et al., 2010; Masel and Trotter, 2010). Because robustness buffers mutational effects, it may seem to be the antithesis of evolvability; however, robustness and evolvability may instead positively correlate (McBride et al., 2008; Draghi et al., 2010). For example, relatively robust proteins have greater structural stability; the effects of mutations that destabilize surface residues do not disrupt the core fold in robust proteins, providing an advantage for evolution of novel secondary functions (Bloom et al., 2006; Tokuriki et al., 2008; Tokuriki and Tawfik, 2009). More generally, theory shows that genetically robust populations contain large neutral networks of genotypes that span broad genotypic space, affording greater access to novel phenotypes following mutation, and allowing relatively robust populations to be more evolvable (Wilke and Adami, 2003; Draghi et al., 2010; Wagner, 2011). This link between evolvability and robustness is experimentally shown for RNA secondary structures and proteins, but rarely for evolving biological populations (Bloom et al., 2006; Elena and Sanjuán, 2008; Draghi et al., 2010).

Empirical studies demonstrating the relationship between robustness and evolvability at the level of populations are difficult for two main reasons: firstly, it can be difficult to identify or construct populations that differ in robustness (though see Montville et al., 2005; Sanjuán et al., 2007; Coleman et al., 2008) and secondly, the timespan necessary to conduct evolution experiments is often very long (Blount et al., 2008; Kawecki et al., 2012). RNA viruses offer an experimental system that can overcome these problems (Elena, 2012). These viruses have a high mutation rate and are easily cultured in the laboratory so the effects of differences in genetic robustness can be studied in a short timespan (Montville et al., 2005; Sanjuán et al., 2007). Furthermore, the small genome sizes of RNA viruses offer the possibility of identifying the specific genetic architectures leading to robustness and how they affect evolvability. Reverse genetic techniques allow large-scale manipulations of the genetic code of viruses, such as switching codons whilst leaving the amino acid sequence intact. Codon switching can increase the percentage of mutations that are non-synonymous, thereby decreasing the number of neutral neighbors and reducing the robustness of a virus population (Lauring et al., 2012).

An alternative way to manipulate robustness in viruses is by varying coinfection level (Montville et al., 2005; Gao and Feldman, 2009). Coinfection allows for complementation, where the effects of harmful mutations are buffered because viruses with deleterious or inactive proteins can be complemented by a coinfecting virus with the beneficial or active protein (Froissart et al., 2004; Aaskov et al., 2006; Gao and Feldman, 2009). Thus, passaging virus populations under high levels of coinfection (and hence, complementation) should reduce selection to maintain robustness at the level of an individual virus, because the environment (coinfection) provides the mutational buffering. Montville et al. confirmed this idea by evolving three populations of the dsRNA bacteriophage ϕ6 for 300 generations at high vs. low multiplicity of infection (MOI), the ratio of infecting viruses to cells (Montville et al., 2005). When clones from the populations were used to found lineages subjected to mutation accumulation (successive bottlenecking that causes mutations to fix via drift), it was revealed that the high-MOI-evolved viruses showed greater variance in the fitness effects of accumulated mutations (reduced robustness) and these populations were termed “brittle.” In contrast, lineages founded by clones isolated from low-MOI-evolved populations showed lesser variance in fitness effects of accumulated mutations, defining these populations as “robust.” Consistent with these findings, Dennehy et al. later showed that greater sequence diversity existed in the low-MOI populations compared to their high-MOI counterparts (Dennehy et al., 2013). This observation suggested that the more robust, low MOI populations had greater genetic variation because they contained a larger neutral network of genotypes.

To test whether robustness imparted an evolvability advantage in phage ϕ6, McBride et al. used clones from the robust and brittle populations to found lineages that were subjected to a novel environment: strong selection pressure (high mortality) caused by periodic exposure to 45°C heat shock (McBride et al., 2008). After passaging the populations through 10 rounds of selection (5 min heat shock, interspersed by 5 generations of growth under normal conditions), improved thermotolerance was observed. However, lineages founded by clones from the robust populations showed a significantly greater increase in thermotolerance, on average, than those founded by clones from the brittle populations. This result indicated that robustness could enhance evolvability, at least in the particular novel environment. However, we note that other virus studies have not always found a positive relationship between evolvability and robustness (Cuevas et al., 2009; Tokuriki et al., 2009).

If viral populations exist in neutral networks, it may be problematic to test the link between robustness and evolvability using populations founded by individual clones (McBride et al., 2008). That is, a clone may not have enough time over the course of a short-term selection experiment to explore the entire neutral network, reducing any differences between small and large networks. Furthermore, studies have shown that rare variants within a population can disproportionately affect evolutionary trajectories (Blount et al., 2008). The current study addresses these caveats by harnessing the same study system to examine whether robustness aids adaptation to the novel environment, but employing genetically-variable population samples (rather than clones) to found test lineages. Similar to McBride et al. (2008), we measured thermotolerance as the key phenotypic change to assess whether robustness enhances evolvability. However, in the current study we monitored phenotypic changes over time (adaptive trajectories) due to the specific prediction that larger neutral networks should foster better access to key mutations that speed adaptation. In addition (unlike the former study), we measured molecular changes via consensus genome sequencing of experimentally evolved populations, to identify whether different beneficial mutations fixed in robust vs. brittle populations.

## Materials and methods

### Strains and culture conditions

**Figure 1:**
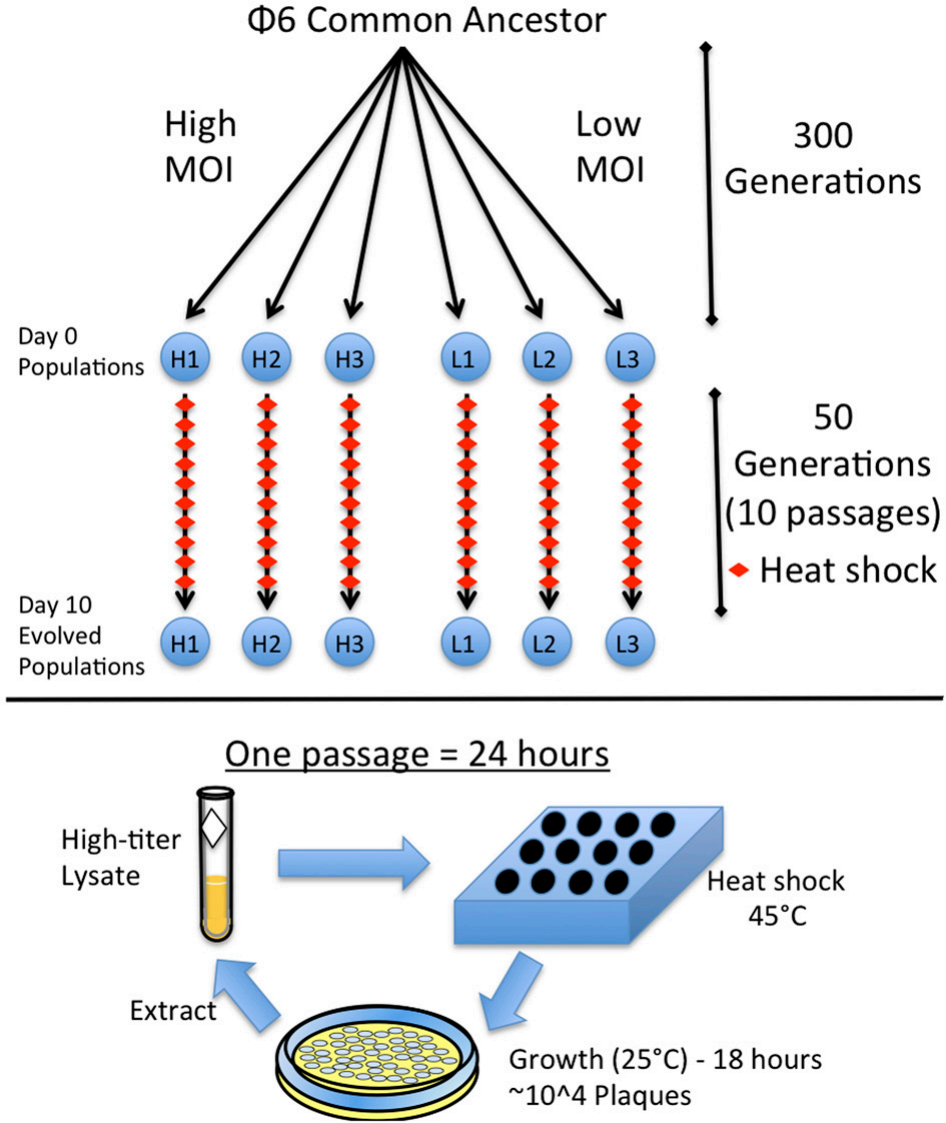
**Experimental design**. Ancestral Φ6 was split into six populations and evolved for 300 generations at high (H1, H2, H3) or low (L1, L2, L3) Multiplicity of Infection (MOI) as described in Montville et al. (2005). These six populations were then evolved for 10 days with a heat shock each day. A single passage consists of heat shocking a diluted viral lysate in soft agar and plating the virus to obtain a plate with ~10,000 plaques. The plates are grown overnight and the virus is then extracted to obtain a high-titer lysate.

A single colony of *Pseudomonas syringae pv. phaseolicola* (American Type Culture collection #21781) was taken from a source plate each day and cultured overnight at 25°C in 10 ml of Luria Broth (LB). The six phage populations of ϕ6 used in this study were previously described (Montville et al., 2005). Three populations were previously evolved for 300 generations under low multiplicity of infection (MOI = 0.002) and characterized as robust: L1–L3 (strains #PT578-PT580); whereas, three otherwise identical populations were evolved at high multiplicity (MOI = 5) and deemed brittle: H1–H3 (strains #PT581-PT583) (Figure 1). Viruses were grown at 25°C in 3 ml of top agar (0.7% agar) containing 200 ul of an overnight bacterial culture, overlaid on LB agar plates (1.5% agar). Lysates were made by removing the top agar, centrifuging in 3 ml of LB and filtering (0.22 μm filter, Millipore) to remove bacteria. Viral lysates were stored in glycerol/LB mixture (2:3 by volume) at −20°C.

### Serial passage with heat shock

To heat shock a virus population, 50 ul of a diluted viral lysate was mixed with 3 ml of top agar and placed in a heating block at 45°C for 5 min; then, 200 ul of overnight bacterial culture was added to the lysate and immediately plated as described above. Different dilutions of the virus lysate were heat-shocked and plated to ensure a resulting plate containing ~10^4^ plaques, which constituted a controlled bottleneck size of the evolving virus population. Lower dilutions with countable numbers of plaques confirmed the bottleneck size was ~10^4^ and non-heat-shocked control dilutions were also plated alongside for comparison, to ensure that heat shock was causing virus mortality. After 24 h, the 10^4^ plaques created a “lacy lawn” (highly overlapping plaques), which yielded an extremely high-titer lysate. The passage was repeated by using the fresh lysate and naïve (non-coevolving) bacteria. Each population was subjected to this daily passage for 10 total days, which was equivalent to 50 generations (5 generations per day) (Turner and Chao, 1998) with 5 min heat shock imposed every fifth generation (Figure 1). A sample of each lysate (evolving population) was frozen at each daily passage.

### Sequencing

High-titer lysates were created of the populations to be sequenced and RNA extracted using QIAamp Viral RNA minikits (Qiagen). RNA was converted into cDNA using SuperScript II (Invitrogen) and then used as template for PCR (primers available on request.) Sanger Sequencing was performed by the Yale Science Hill DNA Analysis Facility. Sequences were manually inspected and analyzed using CLC DNA Workbench 6. Sequences are available in Genbank under the following accession numbers: KF996287-KF996304.

### Survivability assay and thermal niche analysis

75 ul of diluted viral lysate was placed in a PCR tube and heated for 5 min at a test temperature (42.5–47.5°C) in a pre-heated Eppendorf Thermocycler. A 50 ul aliquot from the heat shocked lysate was plated, alongside an otherwise identical control plate (“mock” heat shock: 5 min incubation at 25°C). Survivability fraction was calculated as the number of plaques formed following heat shock divided by those formed on the control plate.

## Results

### Thermotolerance of initial populations

**Figure 2:**
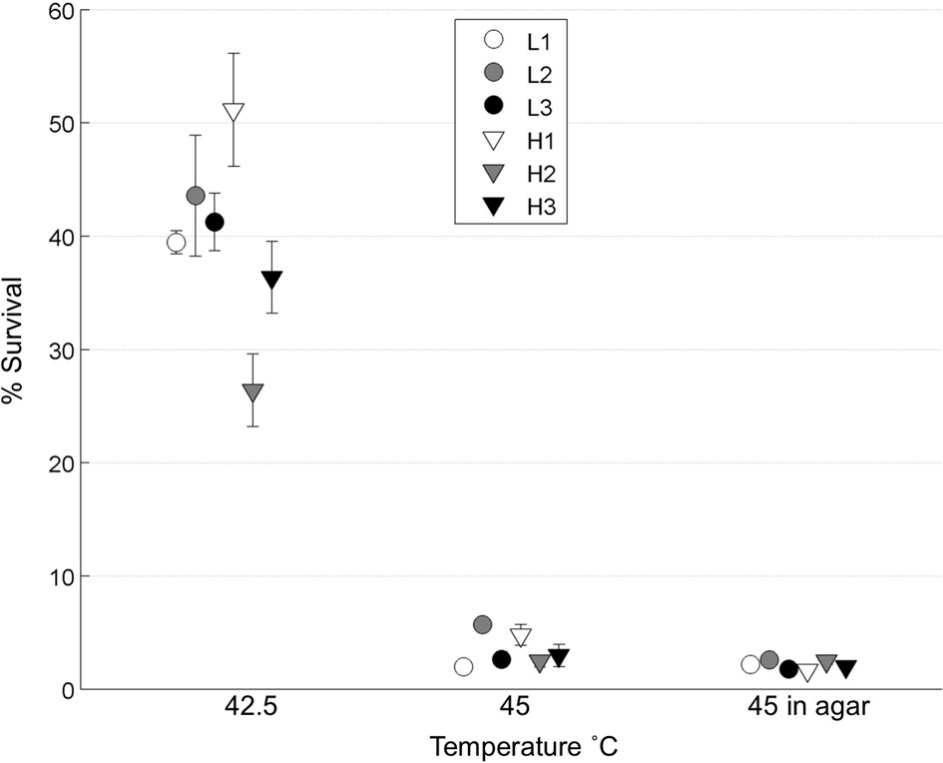
**Initial thermotolerance**. The initial survival of the day 0 populations was calculated following heat shock of viral lysates in a thermocycler at 42.5 or 45°C (*n* = 4), and in soft agar at 45°C (*n* = 5). Error bars show standard error and data points are offset for clarity.

We tested whether the low-MOI evolved “robust” populations (L1, L2, L3) and high-MOI evolved “brittle” populations (H1, H2, H3) initially differed in survival following heat shock. To do so, each population was subjected to replicated (*n* = 4 or 5) 45°C heat shock assays in top agar, as well as assays in a thermocycler at two different temperatures: 42.5 and 45°C. Results (Figure 2) showed no statistical differences in the initial thermotolerance of robust and brittle populations [Two-Way ANOVA, *F*_(1, 12)_ = 0.25, *p* = 0.63]. The observed low survival confirmed that periodic 45°C heat shock would create a strong selective pressure, and that initial survival did not differ according to prior ecological history (low vs. high MOI experimental evolution).

### Faster thermotolerance adaptation in robust populations

To test how robustness affected evolution of thermotolerance, we evolved the six populations in a novel heat shock environment using a constant bottleneck size of 10,000 pfu (plaque forming units). As population size is correlated with speed of evolution (Szendro and Franke, 2013), it was important to maintain equivalent population size among the robust and brittle experimental populations. The bottleneck size of 10,000 individuals was large enough to permit extensive genetic diversity, whilst allowing ~5 generations of evolution each day as the populations grew to their maximum size that preceded the bottleneck (Turner and Chao, 1998). Previous experiments showed rapid evolution in phage ϕ6 populations when cultured at sizes comparable to our study (Burch and Chao, 1999). Montville et al. (2005) showed no fitness differences between robust and brittle populations used to initiate the current study, which suggests that these populations experienced comparable generation numbers during the prior experimental evolution, and that no pre-existing fitness bias favored an evolvability advantage in our experiment.

**Figure 3:**
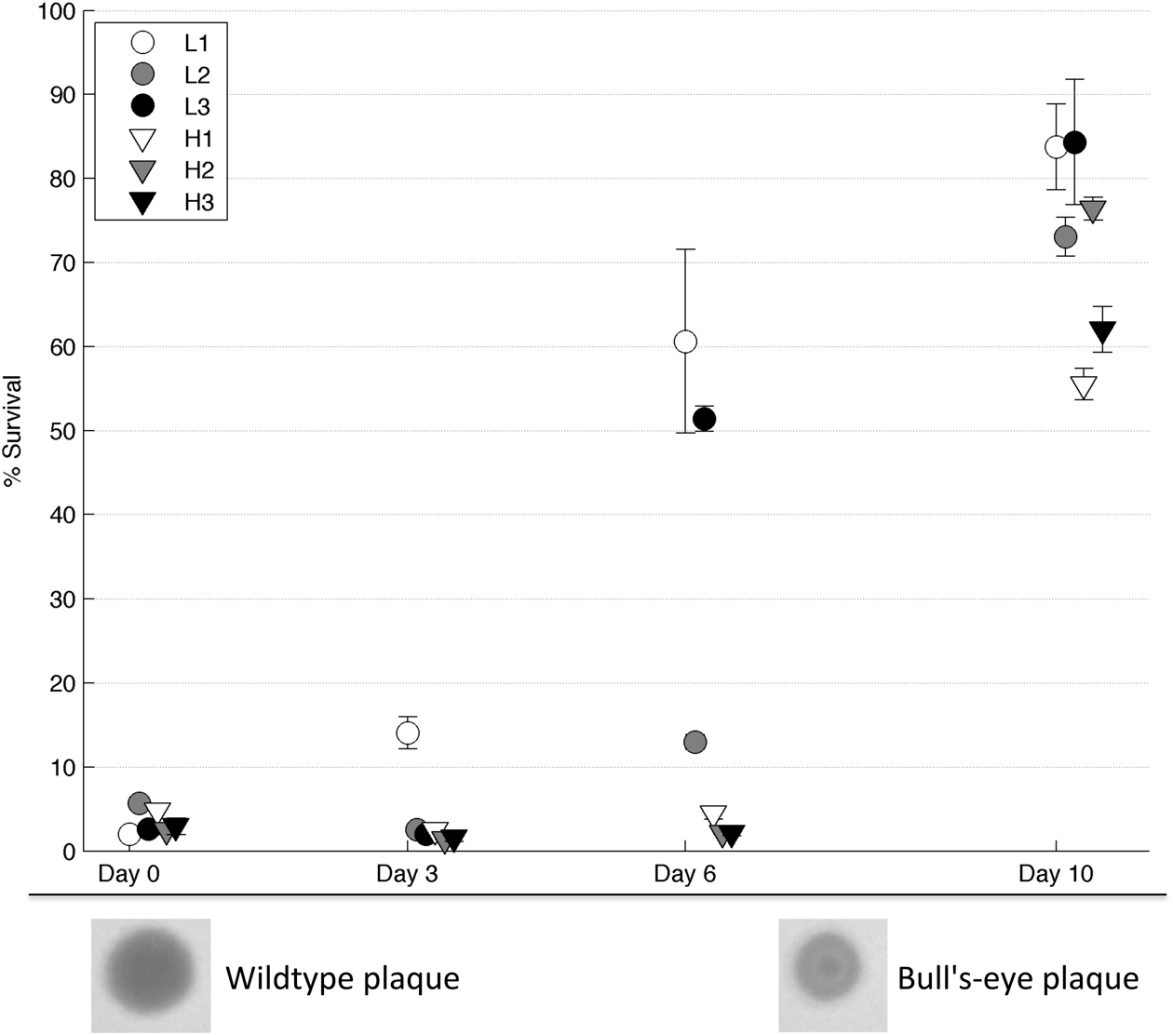
**Survival on intermediate days**. Populations from day 3, 6, and 10 were heat shocked and the percent survival was measured (*n* = 4). Error bars show standard error and data points are offset for clarity. Below the graph, a wildtype (clear) plaque is shown on the left and a bull's-eye plaque on the right.

Following 10 days of evolution, all of the populations showed increased thermotolerance compared to their founding (day 0) population (2-tailed paired *t*-test, *T* = 13.5, *d.f.* = 5, *p* < 4^*^10^−5^, see Figure 3). As described in Dessau et al. (2012), phage ϕ6 strains with improved thermotolerance often show a “bull's-eye” plaque phenotype when grown on agar under normal conditions of 25°C (see Figure 3). Three of the six evolved populations (i.e., L1, L3, H2) showed apparent fixation of the bull's-eye phenotype, which was the only morphotype observed on day 10 plate dilutions of these populations. In contrast, two of the evolved populations (i.e., L2, H1) were polymorphic for bull's-eye plaques, with L2 showing ~50% bull's-eyes and H1 producing ~10% bull's-eyes. Last, a single evolved population (H3) showed almost entirely clear (wildtype phenotype) plaques at the end of the experiment.

To test whether the rate of phenotypic adaptation differed between robust and brittle populations, samples from each population at intermediate days were tested for thermotolerance. Results (Figure 3) showed that robust population L1 had increased in thermotolerance after 3 days, whereas all other populations had not. After 6 days, all of the robust populations showed an increase in thermotolerance, compared to a minimal such increase in the brittle populations; this group-wise difference was statistically significant (2-tailed *t*-test, *T* = 3.74, *d.f.* = 4, *p* = 0.02). The bull's-eye phenotype allowed a visual estimate of the penetrance of thermotolerance mutations. Bull's-eye plaques were first seen in population L1 after the second passage and in populations L2 and L3 shortly thereafter. The bull's eye phenotype was apparently fixed in population L1 by the sixth passage. The bull's-eye phenotype reflected the observed thermotolerance data in population L1: a small increase in thermotolerance by day three coincided with low frequencies of observed bull's eyes, while the higher increase in thermotolerance by day 6 coincided with bull's eyes as the only visible phenotype. To confirm that there were no bull's-eyes present in the original population, we heat shocked the day 0 L1 population and plated multiple dilutions to visualize individual plaques; here we observed no bull's-eyes after screening ~10,000 plaques.

### Molecular evolution

**Figure 4:**
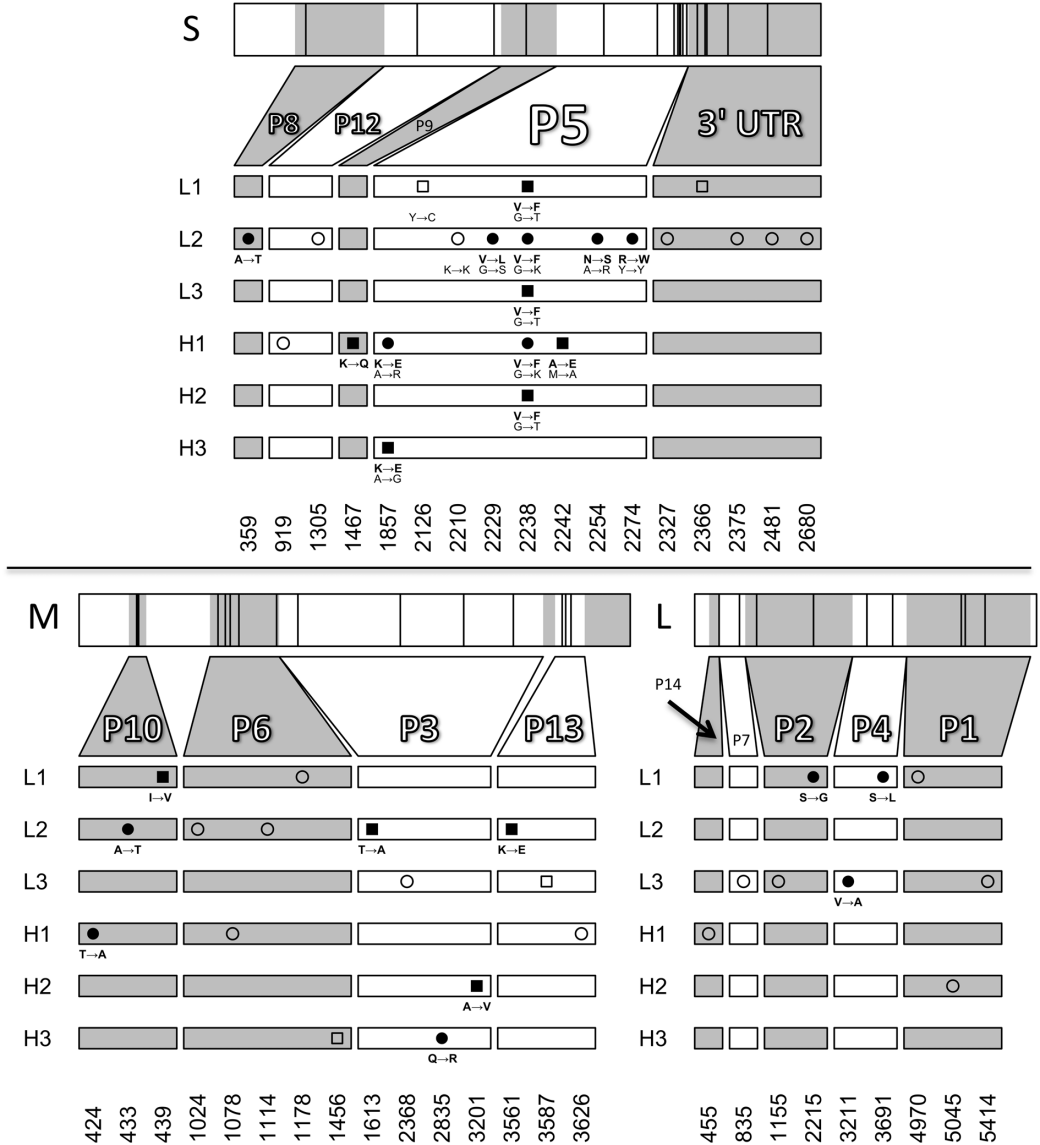
**Sequencing of evolved populations**. The locations of novel mutations on the small, medium, and large RNA segments are plotted for day 10 populations. Squares and circles represent fixed mutations and polymorphisms respectively. Amino-acid changes are shown for non-synonymous mutations (filled symbols) and nucleotide changes are shown for all mutations in P5. Open symbols represent synonymous changes or mutations in untranslated regions. Mutations at positions 2210 and 2274 in the small segment were pre-existing polymorphisms present in the starting population. Positions 2126 and 2242 on the small segment and 439, 1613, 3201, and 3561 on the medium segment are fixed mutations resulting from losses of polymorphism.

The genome size of phage ϕ6 is ~13 kb and consists of three dsRNA segments (Small, Medium and Large). To investigate whether there were differences among the evolved populations in mutations leading to thermotolerance, we sequenced the endpoint (day 10) evolved populations. The consensus sequences revealed several polymorphisms but few fixed protein changes across the six populations (Figure 4). The only locus that showed changes shared among multiple populations was the gene for protein P5, the viral lysin. One non-synonymous change in P5, G2238T (a valine to phenylalanine mutation), was fixed in three populations (L1, L3, H2) and polymorphic in two other populations (L2 and H1). This mutation was previously shown to increase thermotolerance by stabilizing the enzyme under elevated heat, while simultaneously causing a bull's-eye plaque phenotype, indicating reduced virus growth under 25°C conditions (Dessau et al., 2012). One other substitution in P5, A1857G (Lysine to Glutamic Acid), was also shared across two populations (H1, H3). Population L2 had a pre-existing polymorphism at position 2274 which remained in the evolved population. In addition, L2 showed two other non-synonymous polymorphisms in P5: G2229C and A2254G. Although sequencing showed that the G2238T mutation was not present in H3, bull's-eye plaques were observed at low levels (~1%) in population H3. We chose four bull's-eye plaques from population H3 on days 9 and 10 for sequencing, to test whether the G2238T mutation was present. However, the G2238T mutation was not found in any of the chosen plaques. Instead, we observed two other mutations near the end of gene P5 in population H3 that presumably caused the bull's-eye phenotype.

### Evolved changes in thermal niche

**Figure 5:**
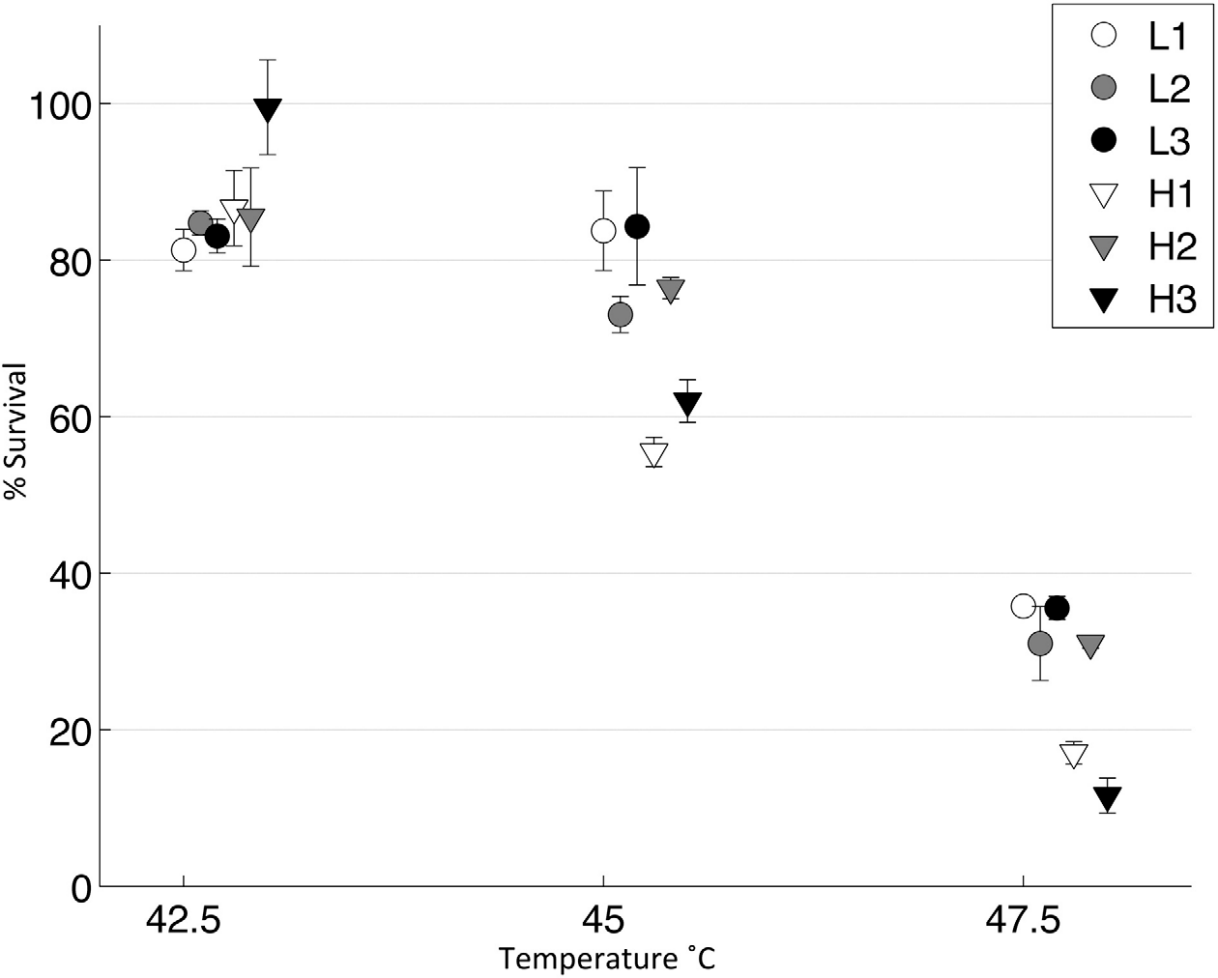
**Thermal niche for evolved populations**. Lysates from each day 10 population were heat shocked in a thermocycler at three different temperatures and then plated (*n* = 3). A control was also plated and percent survival was calculated. Error bars show standard error and data points are offset for clarity.

We tested the survivability of the evolved populations at three different temperatures to approximate a thermal reaction norm for each population (Figure 5). We found no significant difference at 42.5°C, as all populations showed very high survival (2 tailed *t*-test, *T* = 1.36, *d.f.* = 4, *p* = 0.18). However, at 45 and 47.5°C, the robust populations were significantly more thermotolerant than the brittle populations [Two Way ANOVA, *F*_(1, 8)_ = 10.27, *p* = 0.01]; there were no significant differences between the robust populations at these temperatures [Two Way ANOVA, *F*_(2, 12)_ = 2.18, *p* = 0.15].

As key mutations were shared between populations, we wanted to test whether the genetic background affected the phenotype and in particular, how the G2238T mutation affected phenotype because it was shared between both brittle and robust populations. The three populations in which the G2238T mutation was fixed- L1, L3 and H2- showed no significant difference in survival at any of the three temperatures suggesting that the mutation had a similar effect on all three genetic backgrounds. Brittle populations, which did not have the G2238T mutation (H3) or had the mutation in a low percentage of the population (H1) shared an alternative mutation (A1857G) and showed lower thermotolerance at 45 and 47.5°C. The overall significantly lower survival at high temperatures of the brittle populations was caused by populations H1 and H3 and not by population H2, which did not differ from the robust populations.

## Discussion

We subjected three robust and three brittle populations of RNA phage ϕ6 to a novel environment—periodic heat shock selection for 10 days—after which all the populations were observed to increase in thermotolerance. However, the robust populations evolved thermotolerance earlier in the experiment, and were significantly advantaged in thermotolerance relative to most of the brittle populations. Thus, we concluded that robustness tended to promote evolvability, when populations of phage ϕ6 experienced the heat-shock selection conditions imposed in our study.

Theory states that populations containing a larger neutral network in genotype space should be more evolvable, because they can access a greater range of mutations from different points on the network (Wagner, 2008). This increased evolvability could occur either through faster access to key mutations- as there is no need for a permissive primary mutation before a secondary mutation evolves- or through access to a greater variety of mutations with large phenotypic effects. Both explanations relate to the findings in our experiment. All of the robust populations (L1, L2, L3) evolved higher thermotolerance by day 6 than the brittle (H1, H2, H3) populations (Figure 3). The robust populations were able to access a key thermotolerance mutation (G2238T) earlier than the brittle populations, confirmed by their greater increase in thermotolerance by day 6 and by the phenotypic appearance of bull's-eye plaques that are associated with the G2238T substitution (Dessau et al., 2012). It is very unlikely that the G2238T mutation was present in any of the founding populations, as the plaquing phenotype was easily distinguishable and was not observed when founding populations were screened in survival assays (i.e., where genotypes producing bull's eye plaques are strongly positively selected). Rather, bull's-eye plaques were not observed until after day 2 (generation 10) in L1 and even later in other populations.

The only mutations shared by multiple populations were in gene P5 on the Small segment. P5 encodes the viral lysin, a lytic transglycosylase enzyme used by phage ϕ6 to penetrate the bacterial cell wall during entry and exit of the host cell (Mindich and Lehman, 1979; Dessau et al., 2012). The lytic enzyme resides in between the protein shell (P8) surrounding the nucleocapsid and the lipid envelope, but little is known about interactions between P5 and other structural elements of phage ϕ6.

The G2238T mutation in P5 was seen in every population except for H3 and was present at low frequency in H1; in contrast, these two populations had a different mutation in P5: A1857G. It seems likely that this mutation also increases thermotolerance because it occurred following selection in two independent populations, it is a non-synonymous change from a positively to a negatively charged amino acid, and it is the only evolved change in gene P5 of population H3, which showed increased thermotolerance but no bull's-eye phenotypes. The A1857G substitution seemed to be a less-beneficial P5 mutation, because it increased thermotolerance to a lesser degree than the G2238T mutation (cf. Figures 4, 5).

The large number of polymorphisms in population L2 suggested the presence of at least two subpopulations. Intriguingly, L2 had a pre-existing polymorphism in gene P5 at position 2274, which remained in the evolved population. This polymorphism might have a negative epistatic interaction with the G2238T mutation, perhaps explaining why G2238T did not fix in this population. L2 had two other polymorphisms in gene P5 (G2229C and A2254G) that may have evolved because the G2238T mutation was selected against in a subpopulation. This will be the subject of further experiments.

All of the robust populations accessed a more beneficial mutation than population H3, in the evolutionary time allowed. Initially, population H3 had no mutations in the amino acid sequence of gene P5 that could have restricted the evolution of the G2238T mutation, whereas H1 and L2 both had mutations close to this locus, such that negative epistasis may explain why the mutation did not fix in either population. Population H3 showed a synonymous mutation in P5; although this change could not affect the protein structure, it could still be negatively epistatic by altering RNA structure. Brittle populations occupy smaller genotypic networks so it is not surprising that there was variation between populations in which mutations fixed: population H2 was in a network that allowed the evolution of G2238T, whereas population H3 may have been in a network that prevented the evolution of this mutation or promoted a less beneficial mutation. When we sequenced bull's-eye plaques to search for the G2238T mutation in H3, we did not find the mutation. Its absence suggests this highly beneficial mutation could not arise or was selected against in H3. When the G2238T mutation evolved, there was no evidence that the phenotypic effects differed between robust and brittle populations depending on the genetic background, as populations L1, L3, and H2 did not significantly differ in survival across a range of temperatures (Figure 5).

Our results complement and extend those of McBride et al. who showed that lineages founded by robust clones evolved greater thermotolerance than those founded by clones from brittle populations (McBride et al., 2008). We showed that the magnitude change in thermotolerance reported by McBride et al. was likely due to either slower evolution in brittle clones or failure in some clones to find a key mutation. Our results also showed that some brittle populations were able to find the same mutation and catch up to the robust populations, as after 10 days there was no significant difference in phenotypic effect. However, some populations appeared unable to evolve a key mutation altogether. This highlighted the importance of starting with a population as opposed to clones as it demonstrated that in some brittle populations, the loss of intrinsic robustness meant the population had shifted to a less evolvable network. That is, we interpret that 300 generations of evolution at high MOI resulted in the loss of intrinsic (genotypic) robustness, because these high-MOI-evolved populations experienced frequent complementation among coinfecting viruses, an “environmental effect” which should have relaxed selection to maintain individual-level robustness. The resulting evolved brittleness of these populations would cause a concomitant shift to a brittle, less evolvable network. In the current study, initiating experimental evolution with population samples would allow us to test whether such brittle populations were indeed on a less evolvable network, whereas starting the experimental evolution with a single clone could only demonstrate that a single point on the network was less evolvable. Starting with populations also allowed us to test the possibility that rare mutations present in the initial populations could impact the evolutionary trajectory. However, we found no evidence of this in our experiment as all the mutations in P5 occurred *de novo* and were not initially present. The initial diversity within the populations of the current study was necessarily limited by the size of the serial-passage bottleneck (*N* ≈500) they experienced in the study immediately preceding ours (Montville et al., 2005). Although we did not explicitly measure this initial diversity prior to starting the current study, the variation must have exceeded that of a single clone; this differing manipulation of initial variation highlights the key difference between our study and that of McBride et al. (2008), when examining relative evolvability of the populations for increased thermotolerance. Beyond this conservative difference in starting conditions, we note that even higher initial diversity in the founding populations could have led to greater differences between our results and those of McBride et al.

Our results differ from those involving a different RNA virus, Vesicular Stomatitis Virus (VSV), in which no link was found between robustness and evolvability (Cuevas et al., 2009). However, unlike our study, the VSV study founded lineages using clones, rather than populations. It is possible that starting from clones removed initial variation and did not allow the full exploration of the neutral network. Cuevas et al. suggested that in robust clones, beneficial mutations might have reduced phenotypic effect, whereas in our study there was no evidence that beneficial mutations had less phenotypic effect in robust populations. Further work will be necessary to resolve conflicting results for different types of RNA viruses and to assess the generalizability of the link between robustness and evolvability.

The mechanism allowing robustness to affect evolvability in our study remains unclear. The robust populations were shown to harbor greater genetic diversity than the brittle populations (Dennehy et al., 2013), which is consistent with the definition of robustness (i.e., expectation that more genotypes of equal fitness can exist in a robust population than in a brittle one). This greater diversity of genetic backgrounds in a robust population affords increased possibility for *de novo* evolution of positive epistasis with new mutations, and could partly explain the relatively faster rate of thermotolerance evolution in robust populations. There were no easily identifiable permissive mutations in the robust populations that were required before the evolution of thermotolerance, as there were no other mutations seen in multiple populations. However, there were candidate mutations in the brittle populations (as well as a subpopulation of L2) that could have restricted the evolution of the most beneficial mutation G2238T. Although the phenotypic effects were similar between the robust and one of the brittle populations at day 10, the increased thermotolerance of the robust populations by day 6 implies that, under direct competition, the robust populations would have a significant advantage.

One potential problem with our experiment is that genetic robustness and environmental robustness have been shown to be linked, often called plastogenetic congruence, though data is sparse at the level of organisms (Ancel and Fontana, 2000; Meyers et al., 2004; Novella et al., 2013). This means that thermotolerance, a form of environmental robustness, might not be an appropriate trait. We controlled for this by testing initial survival at both 42.5 and 45°C (Figure 2). We found no evidence that the robust populations were pre-adapted to be more thermotolerant as there was no survival difference between robust and brittle populations at 42.5 and 45°C. However, these measurements were taken for the entire population and there may have been individual clones, which were more thermotolerant (Ogbunugafor et al., 2009). Future work could test if our evolvability results are generalizable to other traits.

It is important to study robustness and evolvability using biological populations, as the results may differ when examining more complex systems than experimenting on individual proteins *in vitro*. Our study is one of the first to experimentally examine the effects of robustness on evolvability using populations that differ in robustness, but future work should extend to other viruses. Robustness of viruses might be important in determining many important viral traits such as the ability to switch hosts and the degree to which a virus is pathogenic (Ogbunugafor et al., 2010; Lauring et al., 2012; Remold, 2012). Robustness may also be a determinant of the success of antiviral therapies such as lethal mutagenesis (Bull et al., 2007). We know little about robustness of viruses in natural systems. If *de novo* mutations are required to respond to environmental changes as opposed to selection on standing variation, relatively robust populations of viruses are likely to have an advantage. A better understanding of robustness will hopefully elucidate how organisms respond to environmental challenges and evolve novel functions.

## Author contributions

Daniel Goldhill and Paul E. Turner designed the study, Daniel Goldhill and Angela Lee conducted the experimental evolution, Daniel Goldhill and Elizabeth S. C. P. Williams conducted thermotolerance assays, Daniel Goldhill and Paul E. Turner analyzed the data and prepared the manuscript. All authors read and approved the final manuscript.

### Conflict of interest statement

The authors declare that the research was conducted in the absence of any commercial or financial relationships that could be construed as a potential conflict of interest.
